# Infectious Diseases, Urbanization and Climate Change: Challenges in Future China

**DOI:** 10.3390/ijerph120911025

**Published:** 2015-09-07

**Authors:** Michael Xiaoliang Tong, Alana Hansen, Scott Hanson-Easey, Scott Cameron, Jianjun Xiang, Qiyong Liu, Yehuan Sun, Philip Weinstein, Gil-Soo Han, Craig Williams, Peng Bi

**Affiliations:** 1 School of Public Health, The University of Adelaide, Adelaide 5005, Australia; E-Mails: michael.tong@adelaide.edu.au (M.X.T.); alana.hansen@adelaide.edu.au (A.H.); scott.hanson-easey@adelaide.edu.au (S.H.-E.); scott.cameron@adelaide.edu.au (S.C.); jianjun.xiang@adelaide.edu.au (J.X.); 2 State Key Laboratory of Infectious Disease Prevention and Control, Collaborative Innovation Center for Diagnosis and Treatment of Infectious Diseases, National Institute for Communicable Disease Control and Prevention, Chinese Center for Disease Control and Prevention, Beijing 102206, China; E-Mail: liuqiyong@icdc.cn; 3 Department of Epidemiology, Anhui Medical University, Hefei 230032, China; E-Mail: sun611007@163.com; 4 School of Biological Sciences, The University of Adelaide, Adelaid 5005, Australia; E-Mail: philip.weinstein@adelaide.edu.au; 5 Communications and Media Studies, School of Media, Film and Journalism, Monash University, Clayton 3800, Australia; E-Mail: gil-soo.han@monash.edu; 6 Sansom Institute for Health Research, University of South Australia, Adelaide 5001, Australia; E-Mail: craig.williams@unisa.edu.au

**Keywords:** climate change, urbanization, infectious disease, disease surveillance, challenges, disease control and prevention

## Abstract

China is one of the largest countries in the world with nearly 20% of the world’s population. There have been significant improvements in economy, education and technology over the last three decades. Due to substantial investments from all levels of government, the public health system in China has been improved since the 2003 severe acute respiratory syndrome (SARS) outbreak. However, infectious diseases still remain a major population health issue and this may be exacerbated by rapid urbanization and unprecedented impacts of climate change. This commentary aims to explore China’s current capacity to manage infectious diseases which impair population health. It discusses the existing disease surveillance system and underscores the critical importance of strengthening the system. It also explores how the growing migrant population, dramatic changes in the natural landscape following rapid urbanization, and changing climatic conditions can contribute to the emergence and re-emergence of infectious disease. Continuing research on infectious diseases, urbanization and climate change may inform the country’s capacity to deal with emerging and re-emerging infectious diseases in the future.

## 1. Introduction

China is one of the largest and most populous countries in the world with nearly 20% of the world’s population [1]. Over the last few decades, China has witnessed an unprecedented economic boom with its Gross Domestic Product (GDP) per capita growing from $193 in 1980 to $6092 in 2012 [2]. However, such great economic growth may have been achieved at the cost of the environment and public health programs as compared to other aspects of development including economy, education and technology. One issue is infectious diseases, which continue to impair population health in this populous country [3]. Although infectious diseases only accounted for about 0.89%–1.06% of all deaths in China in 2011 [4], estimates suggest that there are about 7 million cases of notified infectious diseases, such as viral hepatitis, pulmonary tuberculosis, dengue and malaria, occurring amongst the 1.3 billion population, leading to approximately 17,000 deaths each year [4,5]. Tremendous efforts were made to reduce infectious diseases after 1949 [6], but some infectious diseases have rebounded and even increased in some areas since the 1980s [7,8,9,10]. Malaria, for example, has been a serious public health problem in China for many years and great efforts have been made to address the problem. Despite a remarkable decline in its incidence from about 2800 per 100,000 in 1970 to 10 per 100,000 in 1990, malaria has re-emerged in some areas since 2001, e.g., Henan, Hubei and Anhui provinces [11].

Prior to the outbreak of severe acute respiratory syndrome (SARS) in 2003, infectious diseases control and prevention had not been given enough attention [7]. The SARS outbreak started in Guangdong Province in November 2012 and challenged the then Chinese infectious disease control and prevention system. After the SARS outbreak, the Chinese government invested heavily in disease control and prevention [12]. As a result, there have been great improvements in public health infrastructure and public health workforce with new laboratories built, a surveillance system upgrade and increases in numbers of public health professionals [12,13].

The recent outbreak of Ebola virus disease in West Africa in 2014 has drawn worldwide attention [14], and accordingly, public health professionals in China have estimated the risk of imported cases and proposed pro-active disease control and prevention actions to minimize the risk of the transmission of the disease to China [15]. This is partly because China has a close trade relationship with Africa involving a frequent flow of travelers [16,17,18]. Public health authorities have been made aware of the need to continuously strengthen capacity to manage current infectious disease control systems to cope with possible emerging and re-emerging infectious diseases challenges.

China is an economically, geographically and climatologically diverse country with a huge population which may be the epicenter of new diseases and strains [19]. However, it remains uncertain how well current disease prevention and management system can sufficiently respond to future challenges of emerging and re-emerging infectious diseases, which may be exacerbated by the rapid process of urbanization, high numbers of migrant workers, and the impacts from a changing climate. In this light, this paper aims to examine China’s capacity to manage infectious diseases in the future, especially in terms of disease surveillance and the likely impacts of two important issues affecting infectious disease trends—increasing urbanization and climate change.

## 2. Disease Surveillance

A timely disease surveillance system is essential for detecting, reporting and monitoring cases and outbreaks of infectious diseases. The existing public health system in China has been equipped with a comprehensive web-based real-time disease surveillance system built into a network of Centers for Disease Control and Prevention (CDC) at the national level, provincial level, prefectural level and county level. China’s disease surveillance system enables 98% of counties and higher-level hospitals and 88% of township hospitals to have direct connection to the CDC disease reporting system [20,21]. If a notifiable infectious disease case is diagnosed and laboratory confirmed, the case will be reported to the web-based real-time surveillance system, the public health officer, the CDC and the health department, who will take responsibility to investigate the case when necessary [22]. Laboratory diagnostic capacity has also been strengthened in recent years [7]. However, the capacity of this system to detect and verify emerging/re-emerging infectious diseases and rapidly respond to future disease outbreaks still has room for improvement [16,23,24]. The status of the overall infectious disease surveillance infrastructure, health workforce and the underreporting of infectious diseases in some areas of China are of concern.

Firstly, previous research has demonstrated that the overall infectious disease surveillance infrastructure was underfunded in China, especially in poor and rural areas [7,22,23]. Although China has risen to become one of the largest economic powers in the world over recent decades, its health care investment is out of pace with its rapid economic growth [25]. Compared with other countries in the world, China ranks among the poorest in terms of public financing for health care [25]. Consequently, the lack of funding in public health has resulted in limitations in technical capacity and insufficient human resources in disease control and prevention to meet the challenge of potential emerging and re-emerging infectious diseases outbreaks [23]. Whilst China’s comprehensive county-to-national level network CDC surveillance and reporting system has been reported as being one of the best in the world [7], greater effort is required to improve the infectious disease surveillance infrastructure. In urban areas infectious disease surveillance infrastructure has received, and continues to receive, financial support from local governments. However, maintenance and update of disease surveillance in county-level rural areas has relatively been neglected, leading to a regional imbalance between urban and rural areas [7]. Furthermore, despite the relative supremacy of urban disease surveillance infrastructure over its rural counterpart, it still has some limitations. In urban areas, for instance, most public health resources are concentrated in tertiary hospitals and central public health departments, while disease surveillance infrastructure in community-level health service centers needs to be strengthened. Better pathogen-based surveillance infrastructure for disease detection in urban areas and a more rapid response to infectious disease outbreaks is needed to ensure the risks of transmission are minimized in areas of high population density [16]. It is imperative, therefore, that initiatives and actions to maintain and update rural disease surveillance infrastructure be taken, whilst concomitantly reinforcing and optimizing urban disease surveillance infrastructure.

Secondly, the health workforce in some rural areas remains poorly trained and unmotivated [7]. In rural and poor areas there are relatively few health workers who have been professionally trained to effectively deal with infectious disease outbreaks. Likewise, few health professionals at provincial or lower levels CDCs have received formal epidemiological training and communication with international counterparts would be limited [7]. The ‘Chinese Field Epidemiology Training Program’ was established in 2001 with the support of the World Health Organization (WHO) and the US Centers for Disease Control (USCDC) with a view to developing and strengthening China’s disease surveillance, field epidemiology, and response capacity. As of 2014, only 194 public health professionals had graduated from the program [26]. However, it still cannot meet the increasing needs for an adequate number of competent and motivated epidemiologists. In addition, for clinical doctors in hospitals, patients are their priority, not public health, which may have an influence on disease surveillance and reporting. One possible reason is that Chinese medical values, cultural norms and heavy workload have shaped clinical doctors who mainly focus on disease treatment rather than prevention [27]. However, in the modern health care system doctors play an important role in infectious disease detection, diagnosis, reporting, treatment and cooperation with local CDC staff. This concern is especially applicable in China, where it is estimated that more than 70% of the health care visits occur at village-level rural clinics. However, local health workers may lack relevant skills in awareness, diagnosis and treatment of rare diseases and emerging infectious diseases [7]. In this light, it is of critical importance to provide systematic and regular training for the health care workforce in CDCs, hospitals and relevant health departments, especially in rural and remote areas of inland provinces.

Thirdly, underreporting of infectious diseases is not uncommon in some areas of China, although in recent years the situation has improved as regulations are now in place. Disease reporting has been listed as an important indicator for evaluating local government’s performance and hospitals are now randomly inspected to monitor the accuracy and quality of infectious diseases reporting. Underreporting may still occur due to various reasons [28]. Moreover, hospital doctors may not always report diseases as required [29]. Effective communication and information sharing between local hospitals and CDCs are critical for addressing the threats to disease control and prevention, yet doctors do not gain any direct and tangible benefits from the provision of disease surveillance information [24]. This communication and information sharing may benefit from effective incentive mechanisms to ensure accurate disease reporting procedures.

In summary, strengthened disease surveillance infrastructure, better trained health workers and further consideration of underreporting are needed to improve the infectious disease surveillance system. The importance of improving the system is further accentuated by the likely impacts on infectious disease transmission of two important issues facing this nation: urbanization and climate change.

## 3. Urbanization

The process of urbanization is of particular concern in modern China. China’s accelerated development during the last few decades has resulted in rapid urbanization with unprecedented large-scale population movement within the country. Reports have indicated 54.7% of the total population lived in urban areas by the end of 2014, while the rate was only 26% in 1990 [30,31]. Rapid urbanization has brought numerous benefits to the country and substantially improved Chinese people’s standard of living on the whole; public health, however, has not progressed to the same extent. This is especially the case for the migrant population, who have made considerable contributions to the country’s economic development and urbanization, but often have less benefits in terms of income, job security, training, education, and health care [32,33,34].

At present, China is estimated to have a migrant population exceeding 221 million [35]. The migrant population, often described as the “floating population”, largely consists of people originating from rural areas to urban areas in search of better economic and social opportunities. The “floating population” constitute a salient and disadvantaged group in China accounting for more than 10% of China’s total population [24]. The migrants are often poor and less educated than the general population in urban areas. They typically live in low quality housing with inadequate sanitation, and have limited access to local health services. The needs of the migrant population have not been taken into full consideration in health care policy-makers’ formulation of relevant policies and regulations, such as health insurance, occupational injury and infectious disease prevention strategies including immunization. Meanwhile, the “floating population” itself is reluctant to spend its already limited discretionary resources on health care. It comes as no surprise that the overall health status of rural-urban migrants is generally lower than that of local urban residents [33]. In particular, migrants can be more susceptible to infectious diseases, such as tuberculosis, human immunodeficiency virus (HIV) and sexually transmitted diseases (STD), as well as vector/food-borne diseases, and are less likely to receive proper treatment or be cured of infectious diseases than local permanent residents [7,34]. Further, children of migrant parents are also shown to have poorer health, lower nutrient intakes and constrained access to education compared to children of local or permanent residents [36]. Additionally, vaccination coverage among migrant children is lower than the rates for children of local or permanent residents [36]. These factors, in addition to the large size, high mobility and poorer health of the migrant population, have the potential to contribute to the spread of infectious diseases, adversely impacting on national health. Access to health care, insurance coverage, education and community support needs to be extended to the migrant population irrespective of residence and employment status.

The rapid process of urbanization has also caused dramatic changes in natural landscapes in China. New buildings, houses, roads, changed river flows and reduced vegetation impact on local ecological systems [37], and consequently influence the transmission of infectious diseases [37,38]. Studies have found that urbanization can impact on vector/rodent-borne infections, as many highly adaptable species in urban areas are important reservoir hosts for vector-borne and rodent-borne pathogens [39,40]. Moreover, accumulated garbage and discarded materials in cities provide food and habitats for rodents and vectors. Where water supplies are inadequate it can be necessary to store water in containers which can become an ideal habitat for mosquito larvae. By contrast, during the rainy season poor drainage systems can also create breeding sites [40,41]. These factors contribute to the emergence and re-emergence of vector/rodent-borne diseases, such as Japanese encephalitis, dengue fever and hemorrhagic fever with renal syndrome (HFRS) [40].

As the process of urbanization continues to accelerate and cities expand in size, there can be negative impacts on public health. High-density living is associated with close contact between humans and increased risks of disease transmission. Additionally, there are higher risks of human-pathogen and human-vector interaction in crowded areas where environmental health and sanitation conditions are poor. The large numbers of migrant workers in the cities constitute a high-risk group who can be more susceptible to local disease agents yet lack health insurance and may delay seeking treatment, thereby increasing the risk of disease transmission to others. These issues see the health care needs in high-density urban areas exceeding current resources. Forward planning needs to consider the implication of urbanization on disease prevention and control in China’s rapidly growing mega-cities.

## 4. Climate Change

Like rapid urbanization, increasing climate change is of great concern to the Chinese government and people [42,43]. Many infectious disease agents such as viruses and bacteria, and vectors such as mosquitoes are influenced by seasonality and changes in temperature, rainfall and humidity [44]. It is widely understood that the process of rapid development and excessive human activities have caused an increase of 0.85 ± 0.2 °C in global mean temperature during the period from 1880 to 2012 [45]. The most recent Intergovernmental Panel on Climate Change report (IPCC Fifth Assessment Report) has predicted an increase of 1.1–6.4 °C in the average global land and ocean surface temperature from 1990 to 2100 and highlighted the significant impact of the rising temperature on infectious disease transmission and human health [45]. In China the rapid economic development during the last few decades has been accompanied by unprecedented environmental changes with a warming climate and more frequent weather-related natural disasters [46]. Studies have found that the average surface temperature in China has risen by 1.3 °C over the past 58 years (1951–2008) [47]. Precipitation from 1900 to 2005 has declined in southern parts of China, but increased by 22%–33% in northwest China [45]. There have also been increased frequency and intensity of extreme weather events such as droughts, landslides, mudslides, and regional and mountain floods [42]. These changes in climate will likely favor the transmission of many climate-sensitive diseases, such as mosquito-borne and rodent-borne diseases by affecting the ecology of the diseases, impacting upon their distribution and the number of disease cases.

Studies in China have indicated an association between climatic factors and the incidence of vector-borne diseases such as malaria and dengue fever, and diseases with rodent reservoirs such as HFRS [48,49,50]. Malaria and dengue are mosquito-borne infectious diseases caused by parasites of the genus *Plasmodium* and dengue viruses, respectively [51,52]. Climatic factors such as temperature and rainfall have been shown to be associated with the incidence of malaria and dengue via a changing impact on the lifecycles of mosquitoes and parasites, and virus replication [53]. Higher temperature can shorten the duration of parasite and virus replication, enhance the reproduction rate of mosquitoes, and increase the frequency of blood feeding and contacts with humans, causing higher incidence of malaria and dengue. However, extremely high temperature may also disrupt mosquito breeding, kill the eggs and larvae, evaporate water in breeding sites and shorten the mosquito survival cycle, leading to a decline in disease incidence [44]. Likewise, rainfall influences the incidence of malaria and dengue. Increased rainfall favors mosquito development by providing aquatic breeding sites, subsequently increasing mosquito populations and the potential incidence of malaria and dengue [44]. Although excessive rainfall and flooding may destroy the existing mosquito breeding sites and restrict disease transmission in the short term, it is likely to create more breeding habitats for mosquitoes and increase disease incidence over time [44].

The incidence of HFRS has also been associated with climatic factors such as temperature, rainfall and relative humidity. Warmer conditions can increase activities of rodents and the rate of rodent reproduction, thus leading to an increase in the incidence of HFRS [54,55,56]. However, hot and dry conditions and lower relative humidity may increase the mortality of rodents, reduce the survival rate of hantaviruses in the reservoir, and thus curb the transmission of HFRS [57,58]. Plentiful rainfall can create an ideally moist environment for rodents and better growth conditions for vegetation that provide a food source, thus leading to an increase in the population size of rodents and contributing to HFRS transmission [55,59,60]. Similarly, increased relative humidity can promote rodent reproduction, infectivity and stability of the virus, and thus increase the incidence of HFRS [50].

The capacity of China’s current infectious disease control and prevention system to address the daunting challenges of emerging and re-emerging climate-related infectious diseases due to climate change is still under researched and investigated. Research conducted among CDC staff in a Provincial CDC about their perceptions of, and attitudes towards, climate change and infectious diseases, found that CDC health professionals had noticed a climate change-related temporal and spatial change in the transmission patterns of certain infectious diseases such as vector-borne diseases, air-borne diseases and water-borne diseases [61,62]. Adaptation measures and actions were also proposed, such as strengthening existing disease surveillance systems and vector monitoring; building CDC capacity in terms of infrastructure and in-house health professional training; developing and improving relevant legislation, policies and guidelines; improving coordination among various government departments; engaging the community in infectious disease research and intervention, and conducting collaborative research in infectious diseases with other institutions [61,62]. However, the two studies were conducted in one province only and similar studies need to be conducted to enhance understanding of the associations between climatic variables and infectious diseases, and to develop appropriate climate change adaptation measures.

## 5. Conclusions

Since the outbreak of SARS in 2003 the public health system in China has improved due to all levels of governments’ increased investment in disease control and prevention. The health status of the Chinese people has also improved [63]. However, infectious diseases still remain a major cause of morbidity and mortality, and this may be exacerbated by rapid urbanization and unprecedented impacts from climate change. Although infectious diseases in China have been significantly reduced over recent decades, China’s current capacity to manage emerging and re-emerging infectious disease outbreaks is facing formidable challenges. A timely, streamlined, well-funded and efficient disease reporting and surveillance system is essential to monitor the threat of potential epidemics, which may not only affect population health in China but may also have wider implications for global health. In order to deal with future infectious disease threats effectively and promptly, comprehensive prevention and response strategies, which integrate a variety of complementary actions and measures, are needed. More research about infectious diseases, urbanization, climate change, and changing demographics needs to be conducted to support efforts to build China’s capacity to control and prevent the spread of emerging and re-emerging infectious diseases in the future.
